# Comparison of conventional corneal crosslinking with the accelerated procedure in progressive keratoconus

**DOI:** 10.1007/s10792-025-03919-2

**Published:** 2025-12-24

**Authors:** Lisa Ramm, Dominik Thomas Trojan, Dierk Wittig, Frederik Raiskup, Ramin Khoramnia, Robert Herber

**Affiliations:** 1 Department of Ophthalmology, Faculty of Medicine and University Hospital Carl Gustav Carus, TU Dresden, Fetscherstraße 74, 01307 Dresden, Germany; 2 Augenaerztliche Gemeinschaftspraxis MVZ, Pirna, Germany

**Keywords:** Keratoconus, Corneal crosslinking, Accelerated protocol, Tomography, Densitometry, Higher-order aberrations

## Abstract

**Purpose:**

To evaluate the long-term efficacy of corneal crosslinking using the standard protocol (S-CXL) and the accelerated protocol (A-CXL) over a period of 8 years.

**Methods:**

This retrospective cohort study included 61 eyes from 61 patients with progressive keratoconus (KC). They were treated with either the S-CXL (3*30, N = 16) or the A-CXL (9*10, N = 45). Visual acuity and corneal parameters, including higher-order aberrations (HOAs), corneal tomography, and corneal densitometry, were recorded prior to the intervention and at four follow-up visits using Scheimpflug tomography. A linear mixed model with Bonferroni correction was used to analyze the longitudinal data.

**Results:**

Anterior corneal curvature parameters were significantly reduced in both groups at 3 and 8 years (all *p* ≤ 0.019). Central and thinnest corneal thickness decreased significantly after 12 and 36 months (*p* < 0.001). Surface variance and vertical asymmetry indices remained unchanged postoperatively, despite the significant reduction at 8 years in the A-CXL group (*p* < 0.001). The greatest increase in corneal optical density was observed in both groups at 6 months in the anterior layer up to a diameter of 10 mm, which roughly normalized at 12 months. HOA coma was reduced in both groups at 36 and 96 months (all *p* ≤ 0.012). In contrast, only the S-CXL group showed a borderline significant improvement in visual acuity after 96 months (*p* = 0.049) which should be interpreted cautiously due to differences in sample size and baseline characteristics. The rate of postoperative complications was comparable in both protocols.

**Conclusion:**

In an 8-year long-term follow-up, both S-CXL and A-CXL successfully halted KC progression. Non-inferiority of the accelerated protocol was demonstrated.

**Supplementary Information:**

The online version contains supplementary material available at 10.1007/s10792-025-03919-2.

## Introduction

Keratoconus (KC) is a chronic, progressive disease that can cause significant visual impairment even at a young age. KC is characterized by bilateral steeping with thinning of the cornea and a reduction in stromal collagen, leading to reduced corneal stiffness [1]. Various therapeutic approaches are available, but the primary goal should be early diagnosis of the disease. In terms of disease progression, it must be stabilized by increasing corneal stiffness to prevent visual impairment. For this purpose, corneal crosslinking (CXL) is the treatment of choice [2, 3].

The original protocol, known as the standard CXL protocol (S-CXL), uses an intensity of 3 mW/cm^2^ for 30-min of irradiation, resulting in a total dose of 5.4 J/cm^2^ [2, 4]. Based on the Bunsen-Roscoe law, it is possible to shorten the treatment time. This is desirable for both patient comfort and workflow efficiency. Several accelerated CXL protocols (A-CXL) with up to 30 /cm^2^ for 3 min have been used, maintaining a total dose of 5.4 J/ cm [2, 5, 6]. Conversely, it has also been shown that the shorter the UV irradiation time, the less effective the treatment^7^. This is related to the limited oxygen diffusion and higher oxygen consumption with high-intensity protocols. A-CXL with an intensity of 9 mW/cm^2^ for 10 min has been shown as effective in corneal stiffening, while maintaining the benefits of accelerated protocols [3, 8].

The aim of this study was to compare the long-term outcomes of both treatment protocols in patients with progressive KC. It should be determined whether both procedures were equally effective in stopping the progression. To this end, visual, tomographic, and aberrometric parameters were considered and compared between the two protocols over a period of 8 years.

## Methods

This retrospective longitudinal study was conducted at the Department of Ophthalmology of the University Hospital Carl Gustav Carus, TU Dresden, Germany. Patients with progressive KC and subsequent CXL were enrolled between 2009 and 2013. Postinterventional data were collected over a period of 8 years. The study protocol adhered to the principles of the Declaration of Helsinki, was approved by the local ethics committee, and registered on clinicaltrials.gov (NCT04251143). Informed consent was obtained from all study participants prior to study entry. KC progression required for study participations was defined as an increase in the maximum keratometry value (K_max_) of at least 1 D within one year. Only one eye per subject was included. Exclusion criteria were previous eye interventions (including CXL in the past), other eye diseases, pregnancy, and inability to consent. Contact lenses were ceased for at least 10 days before each measurement.

Best-corrected visual acuity was determined, and an ophthalmological examination of the anterior segment and fundus was performed. The study measurements were performed using the Pentacam (Pentacam HR, Type 70,900, Oculus, Wetzlar, Germany). To avoid bias in the measurement results due to a device change during the observation period, only subjects for whom all measurements were performed with the same Pentacam were included. This explains the limited number of cases in the S-CXL group, as the A-CXL protocol was primarily used from a certain point in the study period. The surgical procedure has already been described [9]. The CXL settings are summarized in Supplement 1.

The Pentacam examination captures topographic and tomographic data of the cornea. This device uses a high-resolution rotating Scheimpflug camera that generates two-dimensional optical cross-sectional images of the cornea and the anterior segment during a 360° rotation. These images are used to determine the topography of the anterior and posterior corneal surface, for spatially resolved pachymetry, for corneal optical densitometry (COD), and for determining the anterior chamber depth [10, 11].

This study included various corneal tomography parameters, such as the anterior radius of curvature (ARC), which defines the curvature of the anterior corneal surface of a 3 mm zone centered on the thinnest point of the cornea [12], the posterior radius of curvature (PRC), which represents the averaged curvature values of this 3 mm zone, and mean and maximal keratometry (K_mean_, K_max_). In addition, the index of surface variance (ISV) indicates the deviation of the individual corneal radius from the average value. The index of vertical asymmetry (IVA) is a measure of the symmetry between the corneal radius and the corneal meridian. Both values are increased in corneal pathologies [13]. The Pentacam can measure pachymetric values of the center (central corneal thickness (CCT) measured at the apex) and the thinnest point (thinnest corneal thickness (TCT)) [14].

The COD is based on the quantitative determination of corneal light scattering as an indicator of corneal clarity [15]. The Pentacam calculates the optical density within a 12 mm diameter corneal area from the measured corneal backscatter values [16, 17]. The COD values are expressed in grayscale units (GSU). A GSU value of 0 corresponds to maximum transparency, while a value of 100 reflects total opacity. The examined cornea is divided into annular regions from the center to the periphery, and the COD is expressed separately. The 2 mm diameter region centered on the corneal apex is followed peripherally by regions with diameters between 2 and 6 mm and between 6 and 10 mm. Within each annular region, three separate layers are evaluated. The anterior region comprises the anterior 120 μm of the cornea, the posterior layer is 60 μm thick, and the middle layer corresponds to the space in between [16, 17]. In the present study, only the anterior and middle layers in the central region affected by CXL were evaluated.

Higher-order aberrations (HOAs) were also evaluated. The root mean square (RMS) is the mean deviation of all Zernike polynomials from the ideal corneal wavefront. This value corresponds to the standard deviation of the wavefront error of the anterior and posterior corneal surfaces. RMS anterior refers to the anterior corneal surface, RMS posterior to the posterior corneal surface. Coma describes the fact that distant object points can take on a comet shape. This is because the bending of light rays depends on the asymmetry of the optical system at different angles of incidence [18, 19]. In this study, the values of horizontal and vertical coma as well as the root mean square of coma (RMS coma) were determined. The closer the values of RMS anterior, RMS posterior, vertical coma, and RMS coma are to 0, the lower the severity of HOAs.

In addition, postoperative complications were recorded using slit lamp examinations at follow-up examinations after 12 and 36 months.

Excel 2016 (Microsoft Corp., Redmond, Washington, USA) was used for data collection, and SPSS (Version 27, IBM Statistics, New York, USA) was utilized for analysis. Normal distribution was verified using Shapiro–Wilk test and Q-Q plots. Metric data were expressed as mean, standard deviation, and 95% confidence intervals. To compare between two groups, the Student`s t-test was used in cases of normal distribution. For longitudinal data, i.e., data collected before and after treatment, the linear mixed model with Bonferroni correction was used. A p-value < 0.05 was considered statistically significant.

## Results

A total of 61 eyes of 61 subjects were included. Of these, 16 patients received treatment according to the S-CXL protocol and 45 patients were treated according to A-CXL protocol. The S-CXL group comprised 14 men (87%) and 2 women (13%), with a mean age of 23.4 ± 8.3 years. The mean age of the A-CXL group was 28.1 ± 10.9 years. This group consisted of 34 men (76%) and 11 women (24%). The baseline data are summarized in Table 1. A comparison of the tomographic data of both groups reveals some parameter differences. It can be assumed that the S-CXL group included, on average, more advanced KC cases.

**Table 1:** Baseline data of patients with progressive keratoconus. Treatment was performed using corneal crosslinking according to standard protocol (S-CXL) or accelerated protocol (A-CXL). (TCT: thinnest corneal thickness, K_max_: maximum keratometry, ARC: radius of curvature of the anterior corneal surface, PRC: radius of curvature of the posterior corneal surface; mean ± standard deviation (95% confidence interval of the mean))

Parameter	**S-CXL group**	**A-CXL group**	***P*-value**
*Baseline data*			
number of eyes	16	45	-
age (years)	23.4 ± 8.3 (19 – 27.9)	28.1 ± 10.9 (24.8 – 31.4)	0.124
male/female	14/2	34/11	0.316
right/left eye	5/11	30/15	**0.014**
visual acuity (logMAR)	0.4 ± 0.2 (0.3 – 0.5)	0.3 ± 0.2 (0.2 – 0.4)	0.083
*Clinical examination findings*			
Vogt striae	1 (6.3%)	1 (2.2%)	−
Fleischer ring	3 (18.7%)	14 (31.3%)	−
corneal scarring	1 (6.3%)	3 (6.7%)	−
*Tomography*			
TCT (μm)	458 ± 38 (438 – 477)	471 ± 37 (459 – 483)	0.249
K_max_ (D)	59.1 ± 5.5 (56.3 – 61.9)	55.9 ± 5.5 (54.2 ± 57.6)	0.053
ARC (mm)	6.3 ± 0.5 (6 – 6.5)	6.6 ± 0.5 (6.5 – 6.8)	**0.029**
PRC (mm)	4.6 ± 0.5 (4.4 – 4.9)	5 ± 0.5 (4.8 – 5.1)	**0.023**

Bold values indicate the *p*-value < 0.05 is statistically significant

### Corneal tomography

**Fig. 1:**
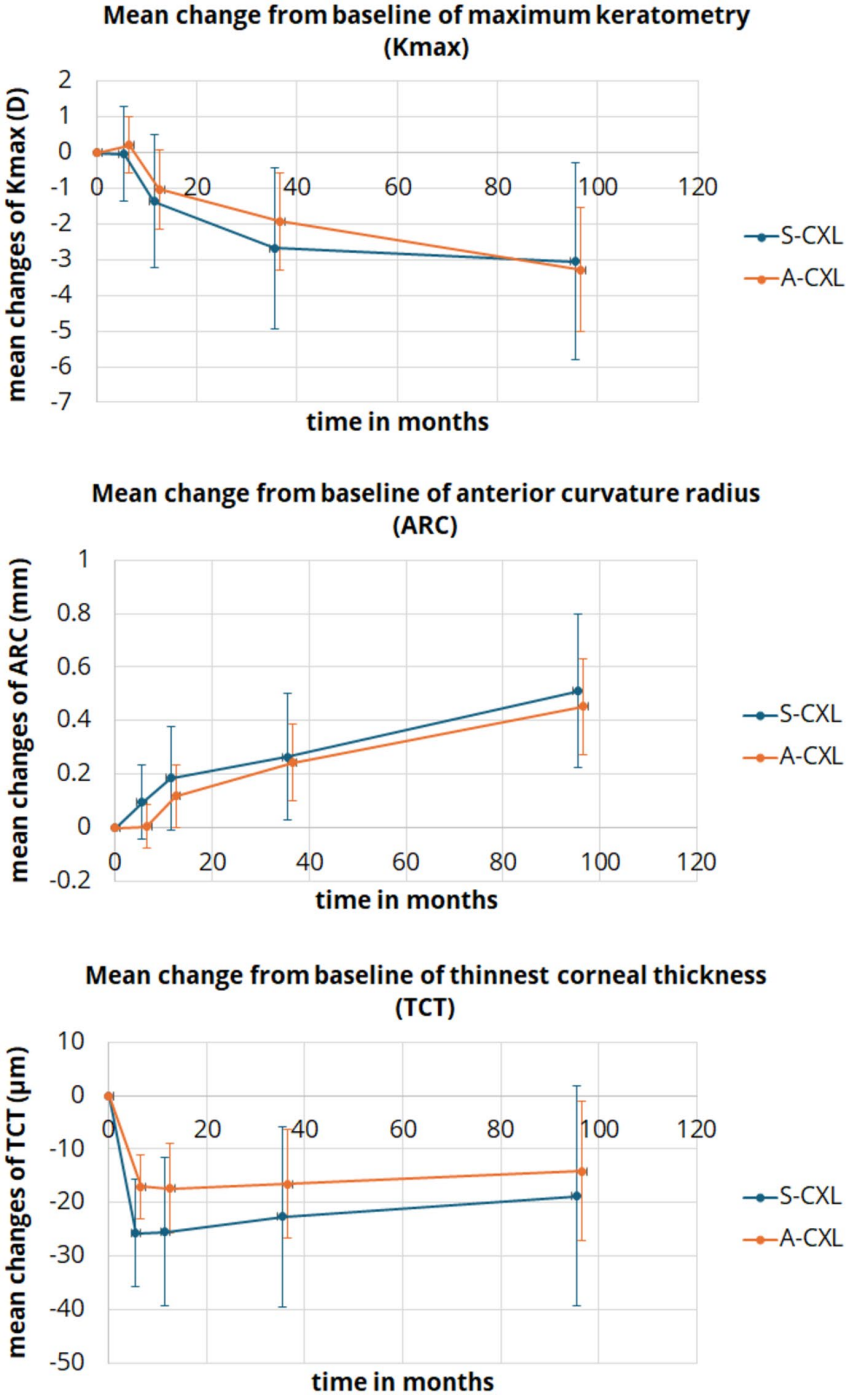
Line plot of mean changes from baseline of tomographic parameters for the standard (S-CXL) and accelerated CXL protocol (A-CXL): maximum keratometry (K_max_), anterior curvature radius (ARC), and thinnest corneal thickness (TCT). Error bars indicate the 95%-confidence interval

**Fig. 2:**
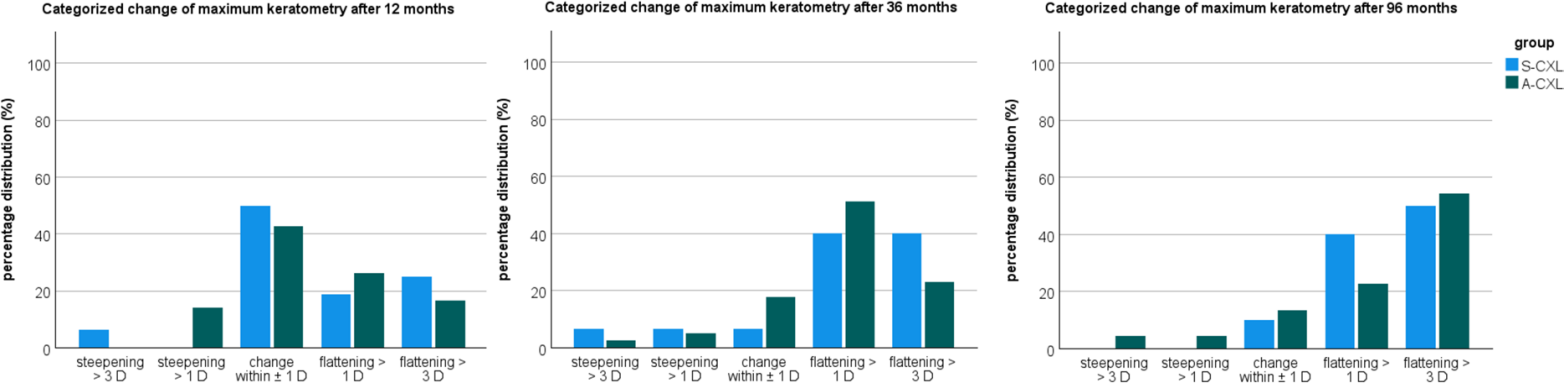
Percentage distribution of categorized change of maximum keratometry (K_max_) for the standard (S-CXL) and accelerated CXL protocol (A-CXL)

The pre- and postoperative tomographic data are presented in Table 2. K_mean_ showed a significant decrease only 96 months after A-CXL (p = 0.047), while K_max_ was significantly lower in both groups at 36 and 96 months compared to baseline (36 months: *p* = 0.009 and *p* < 0.001; 96 months: *p* = 0.019 and *p* < 0.001, Fig. 1). Similarly, ARC (curvature radius) increased gradually over time in both groups (Fig. 1). ISV and IVA were significantly lower only in the A-CXL group at 96 months (*p* < 0.001) and IVA at 36 months (*p* = 0.049) compared to the pre-CXL examination. TCT and CCT differed from baseline at several measurement points in both groups (*p* < 0.05, Fig. 1). Figure 2 shows the categorized change in K_max_, which indicates an increased distribution of corneal flattening after 36 and 96 months compared to 12 months postoperatively.

**Table 2:** Comparison of tomographic parameters in keratoconus patients between baseline and postoperative time points after corneal crosslinking according to standard protocol (S-CXL) or accelerated protocol (A-CXL). (K_mean_: mean keratometry, K_max_: maximum keratometry, ARC: radius of curvature of the anterior corneal surface, PRC: radius of curvature of the posterior corneal surface, ISV: index of surface variance, IVA: Index of vertical asymmetry, TCT: thinnest corneal thickness, CCT: central corneal thickness; mean ± standard deviation (95% confidence interval of the mean))

Parameter		Baseline	6 months	12 months	36 months	96 months	*P*-value			
							Baseline vs 6 months	Baseline vs 12 months	Baseline vs 36 months	Baseline vs 96 months
K_mean_ (D)	S-CXL	47.9 ± 5.2 (46 – 49.8)	48 ± 5.2 (46.1 -49.9)	47.7 ± 5.6 (45.8 – 49.6)	47.5 ± 5.9 (45.6 – 49.4)	46.8 ± 4.4 (44.9 – 48.8)	1.0	1.0	1.0	0.409
	A-CXL	46.8 ± 3.1 (45.7 – 48)	46.9 ± 3.3 (45.8 – 48.1)	46.6 ± 3.2 (45.4 – 47.7)	46.2 ± 3.1 (45.1 – 47.4)	45.9 ± 2.9 (44.7 – 47.1)	1.0	1.0	0.223	**0.047**
K_max_ (D)	S-CXL	59.1 ± 5.5 (56.3 – 61.9)	59.1 ± 5.1 (56.3 – 61.9)	57.7 ± 6.5 (54.9 – 60.5)	56.6 ± 6.8 (53.6 – 59.2)	55.5 ± 5.3 (53.2 – 58.9)	1.0	0.383	**0.009**	**0.019**
	A-CXL	55.9 ± 5.5 (54.2 ± 57.6)	56.1 ± 5.7 (54.4 – 57.8)	54.9 ± 5.6 (53.1 – 56.5)	54 ± 5.7 (52.3 – 55.7)	52.6 ± 5.1 (50.9 – 54.4)	1.0	0.094	** < 0.001**	** < 0.001**
ARC (mm)	S-CXL	6.3 ± 0.5 (6 – 6.5)	6.4 ± 0.5 (6.1 – 6.6)	6.4 ± 0.6 (6.2 – 6.7)	6.5 ± 0.6 (6.2 – 6.8)	6.8 ± 0.7 (6.5 – 7.1)	0.556	0.072	**0.017**	** < 0.001**
	A-CXL	6.6 ± 0.5 (6.5 – 6.8)	6.6 ± 0.5 (6.5 – 6.8)	6.7 ± 0.6 (6.6 – 6.9)	6.9 ± 0.6 (6.7 – 7)	7.1 ± 0.5 (6.9 – 7.2)	1.0	**0.046**	** < 0.001**	** < 0.001**
PRC (mm)	S-CXL	4.6 ± 0.5 (4.4 – 4.9)	4,5 ± 0,5 (4,3 – 4,8)	4,5 ± 0,4 (4,2 – 4,7)	4,5 ± 0,5 (4,2 – 4,7)	4,6 ± 0,4 (4,3 – 4,8)	0.094	0.077	0.336	1.0
	A-CXL	5 ± 0.5 (4.8 – 5.1)	4,9 ± 0,5 (4,7 – 5)	4,9 ± 0,5 (4,7 – 5)	5 ± 0,5 (4,8 – 5,1)	5,1 ± 0,5 (5 – 5,3)	0.055	0.239	1.0	0.063
ISV (D)	S-CXL	8.1 ± 4.1 (6.2 – 10.1)	8.7 ± 3.9 (6.8 – 10.7)	8.5 ± 4.1 (6.6 – 10.5)	7.7 ± 3.9 (5.8 – 9.7)	7.3 ± 4.1 (5.2 – 9.3)	0.109	1.0	1.0	0.998
	A-CXL	6.6 ± 3.7 (5.4 – 7.8)	6.8 ± 3.8 (5.7 – 8)	6.4 ± 4 (5.3 – 7.6)	6 ± 4 (4.8 – 7.1)	5.3 ± 3.3 (4.2 – 6.5)	1.0	1.0	0.148	** < 0.001**
IVA (D)	S-CXL	1.2 ± 0.4 (1 – 1.5)	1.3 ± 0.4 (1.2 – 1.5)	1.2 ± 0.5 (1 – 1.5)	1.1 ± 0.4 (0.8 – 1.1)	1.1 ± 0.6 (0.8 – 1.3)	0.550	1.0	0.104	0.379
	A-CXL	1 ± 0.5 (0.9 – 1.2)	1 ± 0.5 (0.9 – 1.1)	1 ± 0.5 (0.8 – 1.1)	0.9 ± 0.5 (0.8 – 1)	0.8 ± 0.4 (0.7 – 1)	1.0	1.0	**0.049**	** < 0.001**
TCT (μm)	S-CXL	458 ± 38 (438 – 477)	432 ± 42 (413—452)	432 ± 44 (413 – 452)	435 ± 40 (415 – 455)	439 ± 28 (418 – 459)	** < 0.001**	** < 0.001**	**0.002**	0.100
	A-CXL	471 ± 37 (459 – 483)	454 ± 40 (442 – 466)	454 ± 39 (442 – 466)	455 ± 41 (443 – 466)	457 ± 44 (444 – 470)	** < 0.001**	** < 0.001**	** < 0.001**	**0.022**
CCT (μm)	S-CXL	492 ± 36 (473 – 511)	466 ± 34 (447 – 486)	473 ± 40 (453 – 492)	479 ± 40 (460 – 498)	474 ± 30 (454 – 494)	** < 0.001**	** < 0.001**	0.190	0.064
	A-CXL	492 ± 37 (481 – 504)	476 ± 41 (464 – 488)	479 ± 40 (467 – 491)	481 ± 42 (469 – 492)	479 ± 42 (466 -491)	** < 0.001**	** < 0.001**	**0.006**	**0.013**

Bold values indicate the *p*-value < 0.05 is statistically significant

### Corneal densitometry

**Fig. 3:**
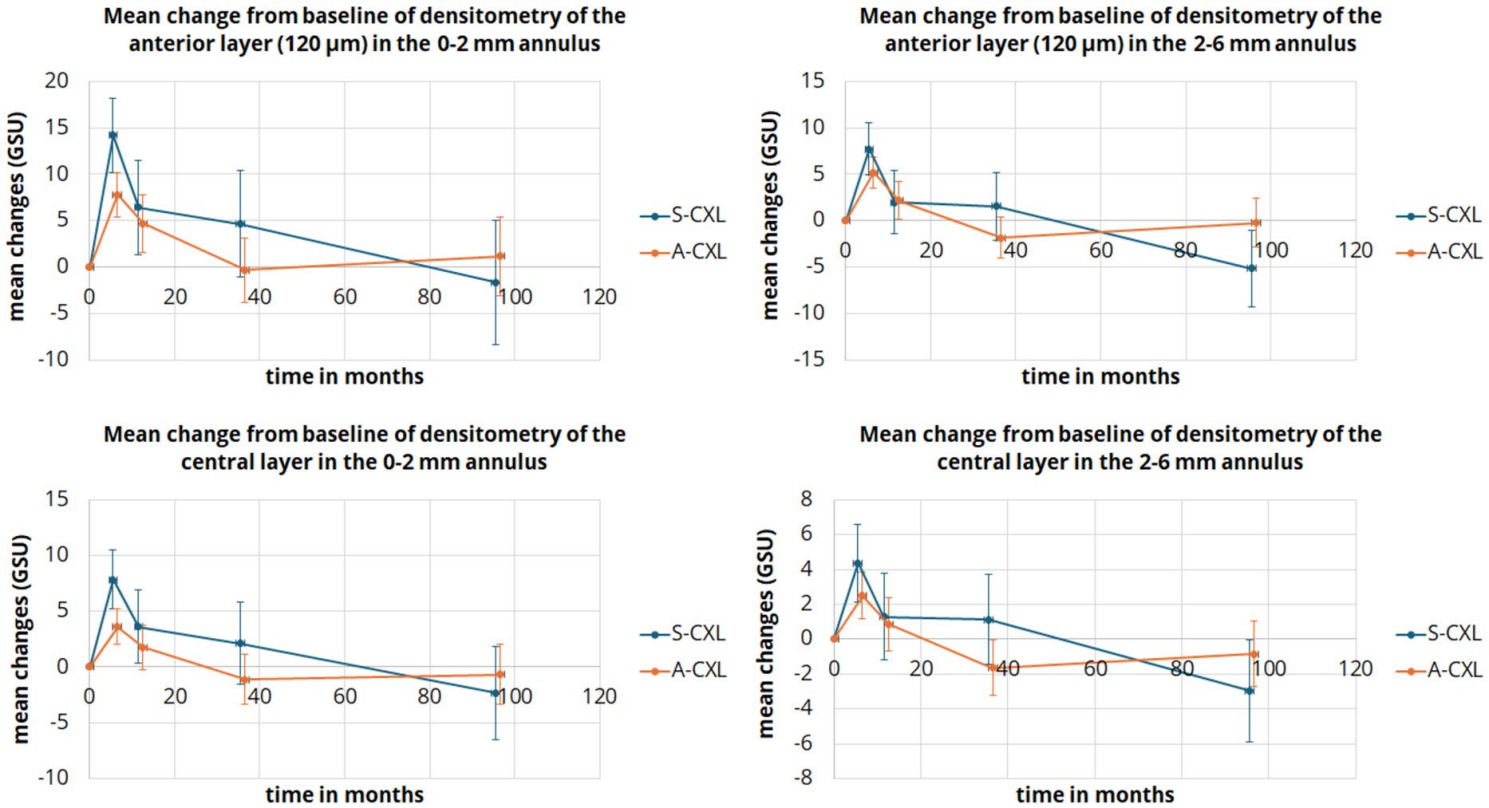
Line plot of mean changes from baseline in anterior and middle layer densitometry values for the standard (S-CXL) and accelerated (A-CXL) protocols in the central and midperipheral annulus. Error bars indicate the 95%-confidence interval

In short, COD increased significantly in both groups after six months in the anterior and middle layers (*p* < 0.001), a change that persisted for up to 12 months. After that, the effect partially normalized at 36 months, with GSU reaching baseline values. For the S-CXL protocol, some annulus and layer values were statistically significantly lower than baseline, potentially due to loss of follow-up or dropouts from follow-up treatments. All corneal densitometry results are summarized in Table 3 and Fig. 3.

**Table 3:** Comparison of densitometry results in keratoconus patients between baseline and postoperative time points after corneal crosslinking according to standard protocol (S-CXL) or accelerated protocol (A-CXL). (Measurement in the anterior and middle corneal layer in the anulus around the corneal center with diameters 0 – 2 mm, 2 – 6 mm and 6 – 10 mm, values given in grayscale units (GSU); mean ± standard deviation (95% confidence interval of the mean))

Parameter		Baseline	6 months	12 months	36 months	96 months	*P*-value			
							Baseline vs 6 months	Baseline vs 12 months	Baseline vs 36 months	Baseline vs 96 months
*Anterior corneal layer*										
0–2 mm (GSU)	S-CXL	32.4 ± 3.3 (29.1 – 35.7)	46.5 ± 7.5 (34.3 – 49.9)	38.8 ± 7.1 (35.6 – 42.1)	37.1 ± 7.2 (33.7 – 40.4)	30.8 ± 11.8 (26.9 – 34.6)	** < 0.001**	**0.004**	0.225	1.0
	A-CXL	29.2 ± 3.7 (27.3 – 31.2)	37.1 ± 7.6 (35.1 – 39)	34 ± 8.5 (32 – 35.9)	29 ± 4.9 (26.9 – 31)	30.4 ± 4.4 (27.9 – 32.9)	** < 0.001**	** < 0.001**	1.0	1.0
2–6 mm (GSU)	S-CXL	27.3 ± 2 (25.5 – 29.2)	35 ± 4.2 (33.2 – 36.9)	29.3 ± 2.6 (27.5 – 31.2)	28.9 ± 4 (27 – 30.8)	22.2 ± 5.9 (19.9 – 24.4)	** < 0.001**	0.943	1.0	**0.005**
	A-CXL	25.5 ± 2.3 (24.4 – 26.6)	30.7 ± 4.7 (29.6 – 318)	27.6 ± 4.4 (26.5– 28.7)	23.7 ± 3.8 (22.5 – 24.8)	25.2 ± 2.7 (23.7 – 26.7)	** < 0.001**	**0.032**	0.204	1.0
6–10 mm (GSU)	S-CXL	21.2 ± 1.8 (19.5 – 22.9)	23.6 ± 2.2 (21.5 – 24.8)	22.1 ± 3 (20.5 – 23.8)	23.1 ± 4.6 (21.4 – 24.8)	17.4 ± 4.9 (15.4 – 19.5)	0.214	1.0	0.953	**0.042**
	A-CXL	21.9 ± 2.6 (20.9 – 22.9)	24 ± 4.5 (23 – 25)	22.2 ± 2.9 (21.2 – 23.2)	19.4 ± 3.5 (18.4 – 20.5)	22.4 ± 3.4 (21.1 – 23.8)	** < 0.001**	1.0	**0.004**	1.0
*Middle corneal layer*										
0–2 mm (GSU)	S-CXL	18.9 ± 1.3 (17 – 20.9)	26.8 ± 8,2 (24.8 – 28,7))	22.5 ± 5.8 (20.6 – 24.5)	21.1 ± 5 (19.1 – 23)	16.6 ± 5 (14.3 – 18.9)	** < 0.001**	**0.022**	1.0	1.0
	A-CXL	17.8 ± 1.6 (16.7 – 19)	21.4 ± 3,9 (20.2 – 22,5))	19.6 ± 4.4 (18.4 – 20.7)	16.7 ± 3.1 (15.5 – 17.9)	17.1 ± 2.4 (15.6 – 18.7)	** < 0.001**	0.140	1.0	1.0
2–6 mm (GSU)	S-CXL	16.2 ± 0.8 (15 – 17.5)	20.6 ± 3,4 (19.3 – 21,9)	17.5 ± 1.9 (16.2 – 18.8)	17.4 ± 3 (16.1 – 18.7)	13.3 ± 2.6 (11.7 – 14.9)	1.0	1.0	1.0	**0.046**
	A-CXL	15.8 ± 1.3 (15 – 16.5)	18.3 ± 3,3 (17.5 – 19)	16.6 ± 3.7 (15.8 – 17.4)	14.1 ± 2.1 (13.3 – 14.9)	14.9 ± 1.6 (13.8 – 16)	1.0	1.0	**0.036**	1.0
6–10 mm (GSU)	S-CXL	14.4 ± 1.7 (12.9 – 15.9)	15.7 ± 2,2 (14.2 – 17,2)	15.3 ± 3.1 (13.7 – 16.8)	16.6 ± 4.4 (15.1 – 18.2)	13.1 ± 3.3 (11.3 – 14.9)	0.711	1.0	0.221	1.0
	A-CXL	15.3 ± 2.4 (14.4 – 16.2)	16.6 ± 4 (15.7 – 17,5)	15.5 ± 2.7 (14.6 – 16.4)	14 ± 2.6 (13.1 – 15)	15.9 ± 3.3 (14.8 – 17.1)	**0.022**	1.0	**0.005**	1.0

Bold values indicate the *p*-value < 0.05 is statistically significant

### Higher-order aberrations

**Fig. 4:**
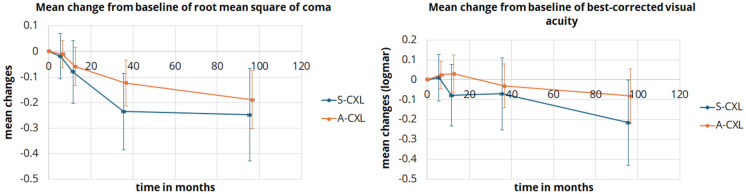
Line plot of mean changes from baseline of root mean square of coma and visual acuity for the standard (S-CXL) and accelerated (A-CXL) protocols. Error bars indicate the 95%-confidence interval

In both groups, HOAs did not differ significantly from baseline in the first examinations 6 and 12 months after CXL. RMS anterior and RMS anterior & posterior differed significantly only in the A-CXL group at 36 and 96 months after CXL (36 months: p = 0.02 and p = 0.049; 96 months: *p* = 0.002 and *p* = 0.016). Both vertical coma and RMS coma (Fig. 4) decreased significantly in both groups at 36 and 96 months compared to baseline (all *p* ≤ 0.012). The comparison of HOAs is shown in Table 4.

**Table 4:** Comparison of higher-order aberrations in keratoconus patients between baseline and postoperative time points after corneal crosslinking according to standard protocol (S-CXL) or accelerated protocol (A-CXL). (RMS: root mean square; mean ± standard deviation (95% confidence interval of the mean))

Parameter		Baseline	6 months	12 months	36 months	96 months	*P*-value			
							Baseline vs 6 months	Baseline vs 12 months	Baseline vs 36 months	Baseline vs 96 months
RMS anterior	S-CXL	0.82 ± 0.24 (0.67 – 0.97)	0.88 ± 0.32 (0.74 – 1.03)	0.73 ± 0.26 (0.59 – 0.88)	0.64 ± 0.22 (0.5 – 0.79)	0.62 ± 0.24 (0.42 – 0.78)	1.0	1.0	0.14	0.20
	A-CXL	0.68 ± 0.31 (0.59 – 0.77)	0.66 ± 0.33 (0.58 – 0.75)	0.62 ± 0.33 (0.53 – 0.71)	0.55 ± 0.32 (0.46 – 0.63)	0.48 ± 0.27 (0.38 – 0.58)	1.0	1.0	**0.02**	**0.002**
RMS anterior & posterior	S-CXL	0.75 ± 0.22 (0.61 – 0.88)	0.82 ± 0.38 (0.69 – 0.96)	0.66 ± 0.23 (0.52 – 0.8)	0.57 ± 0.23 (0.44 – 0.71)	0.62 ± 0.17 (0.46 – 0.77)	1.0	1.0	0.15	1.0
	A-CXL	0.6 ± 0.28 (0.52 – 0.68)	0.58 ± 0.31 (0.50 – 0.67)	0.55 ± 0,3 (0.47 – 0.63)	0.48 ± 0.30 (0.4 – 0.56)	0.43 ± 0.25 (0.34 – 0.53)	1.0	1.0	**0.049**	**0.016**
Coma horizontal	S-CXL	0.05 ± 0.37 (− 0.07 – 0.17)	0.04 ± 0.34 (-0.08 – 0.16)	− 0.02 ± 0.33 (− 0.14 – 0.1)	0.01 ± 0.29 (-0.11 – 0.13)	0.03 ± 0.27 (− 0.1 – 0.15)	1.0	0.31	1.0	1.0
	A-CXL	− 0.04 ± 0.21 (-0.11 – 0.03)	-0.04 ± 0.21 (-0.08 – 0.16)	− 0.02 ± 0.21 (− 0.09 – 0.05)	− 0.02 ± 0.18 (− 0.09 – 0.05)	− 0.03 ± 0.13 (− 0.1 -0.05)	1.0	1.0	1.0	1.0
Coma vertical	S-CXL	− 0.64 ± 0.32 (− 0.79 – -0.48)	-0.61 ± 0.29 (-0.77—-0.46)	− 0.52 ± 0.37 (− 0.67—-0.36)	− 0.42 ± 0.26 (− 0.58—-0.27)	− 0.41 ± 0.27 (-0.57—-0.24)	1.0	0.16	**0.003**	**0.012**
	A-CXL	0.6 ± 0.3 (− 0.69—-0.51)	-0.59 ± 0.32 (− 0.68—-0.49)	-0.53 ± 0.33 (− 0.62—-0.43)	− 0.47 ± 0.33 (− 0.56—-0.38)	− 0.42 ± 0.27 (− 0.52—-0.31)	1.0	0.15	**0.003**	** < 0.001**
RMS Coma	S-CXL	0.74 ± 0.28 (0.60 – 089)	0.73 ± 0.2 (0.58 – 0.87)	0.66 ± 0.26 (0.52 – 0.81)	0.51 ± 0.24 (0.36 – 0.66)	0.5 ± 0.25 (0.34 – 0.65)	1.0	0.67	** < 0.001**	**0.001**
	A-CXL	0.64 ± 0.3 (0.55 – 0.72)	0.62 ± 0.33 (0.54 – 0.71)	0.58 ± 0.31 (0.49 – 0.66)	0.51 ± 0.33 (0.42 – 0.6)	0.45 ± 0.28 (0.35 -0.54)	1.0	0.26	**0.001**	** < 0.001**
Spherical aberrations	S-CXL	− 0.05 ± 0.15 (-0.1 – 0)	− 0.03 ± 0.17 (-0.08 – 0.03)	− 0.03 ± 0.12 (-0.08—-0.03)	− 0.01 ± 0.12 (− 0.06—-0.05)	− 0.01 ± 0.13 (− 0.05 – 0.06)	1.0	1.0	0.84	0.58
	A-CXL	− 0.05 ± 0.09 (− 0.08—-0.02)	− 0.05 ± 0.09 (-0.08—-0.02)	− 0.04 ± 0.09 (− 0.07 – -0.01)	− 0.03 ± 0.08 (− 0.06 – 0)	− 0.03 ± 0.07 (− 0.06 – 0.01)	1.0	1.0	1.0	1.0

Bold values indicate the *p*-value < 0.05 is statistically significant

### Visual acuity

**Fig. 5:**
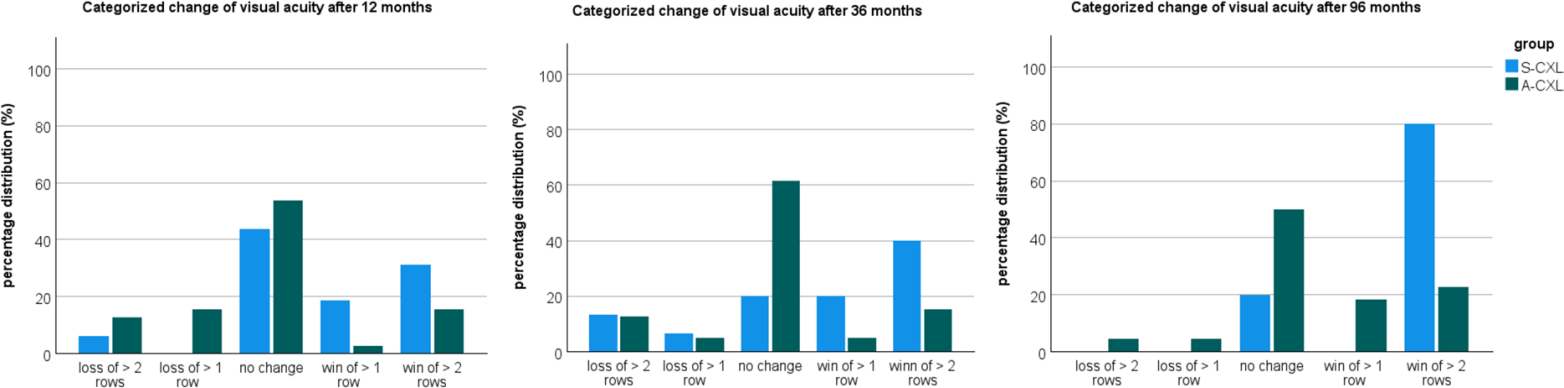
Percentage distribution of categorized change of visual acuity for the standard (S-CXL) and accelerated CXL protocol (A-CXL)

Compared to baseline, visual acuity after CXL showed mild significant change. Only in the S-CXL group there was a slight, but significant increase in visual acuity after 96 months (*p* = 0.049). However, this difference should be interpredet with caution due to differences in sample size and basline data between the study groups. Table 5 and Fig. 4 shows the visual acuity at the follow-up examinations and the respective comparison to baseline. Figure 5 illustrates the categorized distribution of changes in visual acuity. Approximately half of the patients showed no improvement or deterioration in vision after A-CXL. An improvement of more than one or two lines was observed in 20% of patients. After eight years, S-CXL showed an improvement of more than two lines in 80% of patients. However, the results can only be evaluated to a limited extent due to the small patient sample size and comparatively high dropout rate.

**Table 5:** Comparison of best-corrected visual acuity in keratoconus patients between baseline and postoperative time points after corneal crosslinking according to standard protocol (S-CXL) or accelerated protocol (A-CXL). (mean ± standard deviation (95% confidence interval of the mean))

Parameter		Baseline	6 months	12 months	36 months	96 months	*P*-value			
							baseline vs 6 months	baseline vs 12 months	baseline vs 36 months	baseline vs 96 months
Visual acuity	S-CXL	0.4 ± 0.2 (0.3 – 0.5)	0.42 ± 0.29 (0.3 – 0.54)	0.33 ± 0.32 (0.21 – 0.46)	0.34 ± 0.31 (0.22 – 0.47)	0.2 ± 0.13 (0.06 – 0.33)	1.0	1.0	1.0	**0.049**
	A-CXL	0.3 ± 0.2 (0.2 – 0.4)	0.31 ± 0.24 (0.24 – 0.38)	0.32 ± 0.32 (0.24 – 0.39)	0.25 ± 0.21 (0.18 – 0.33)	0.2 ± 0.25 (0.12 – 0.29)	1.0	1.0	1.0	0.943

Bold values indicate the *p*-value < 0.05 is statistically significant

### Complications

Postoperative complications were examined at 12 and 36 months. In the S-CXL group, 50% of patients showed no corneal complications at both examination times. Local scarring occurred postoperatively in 25% of patients, but this did not affect the optical center and had only mild impact on visual performance. In approximately 25% of cases, corneal opacities were visible in slit-lamp biomicroscopy, which are related to the depth of the CXL effect. In the A-CXL group, 44% of patients showed no corneal complications at 12 months. Local scars were present in 11% of patients, while corneal opacities were documented in 36% of patients. One patient developed a keratitis in the early postoperative period (2%, genesis not documented). At 36 months, 49% of A-CXL patients showed no corneal complications, 31% had mild corneal opacities, and 7% showed a corneal scar.

Four patients (25%) in the S-CXL group experienced progression, as determined by consecutive measurements with an increase im K_max_ value of more than 1 D. These patients required CXL retreatment after a mean follow-up of 61.3 ± 23.9 months. The affected patients were generally younger (between 17 and 25 years) and had an advanced stage of KC (more than 55 D of K_max_). Two of all patients in the group were lost to follow-up three and eight years later.

In the A-CXL group, five patients (11%) showed progression. These patients required repeat CXL after a mean follow-up of 46.4 ± 29.1 months. One patient showed a progression of more than 3 D after 96 months, thus indicating a CXL retreatment. Two patients underwent further procedures, including alcohol keratectomy and Descemet membrane endothelial keratoplasty, and were excluded from subsequent follow-up. Sixteen patients were lost to follow-up between three and eight years later.

## Discussion

In both groups, CXL resulted in significant changes in corneal parameters compared to baseline. Tomographic examinations showed an improvement in K_max_ and ARC in the S-CXL group. After A-CXL, these parameters improved, as did K_mean_, IVA, and ISV.

IVA and ISV are parameters of surface variance. Corneal uniformity increases with decreasing values. This was the case for both S-CXL and A-CXL during the follow-up period, but only reached significance for A-CXL at later measurement points. This may indicate a more comprehensive improvement in parameters after A-CXL than after S-CXL, although the A-CXL group had some better baseline values (ARC, PRC).

In contrast, in a previous study, Prinz et al. found no change in K_max_ for both CXL protocols over a 24-month observation period. In a similar study, Nicula et al. reported significantly improved K_max_, IVA, and ISV for both S-CXL and A-CXL up to 7 years after CXL. This study showed no significant difference in K_max_ between the two CXL protocols postoperatively [20]. A significant reduction in K_max_ after CXL has already been shown in various studies [14, 20, 21]. Both the results of Nicula and coworkers as well as the current investigation suggest that A-CXL is non-inferior to S-CXL in this respect. Concerning the changes in corneal thickness, Prinz et al. found a significant decrease in CCT after both S-CXL and A-CXL [22], and Shajari et al. preferred A-CXL in terms of minimum corneal thickness [23]. However, they found no difference in CCT between the groups [23]. On the other hand, in the present study, TCT and CCT increased slightly after the intervention compared to baseline. Although this effect was significant at most follow-up time points, its true relevance is likely limited due to the small change of just a few micrometers. For example, to demonstrate a clinically relevant effect in applanation tonometry, corrections to the measured intraocular pressure are only recommended when changes in CCT occur in increments of 25 µm [24]. This value is not reached in either group, even after 96 months. It should be noted, however, that none of the CXL procedures resulted in further relevant corneal thinning over a long postinterventional period.

COD initially showed a postinterventional increase in OD with both S-CXL and A-CXL. In the middle corneal layer, there were no differences in OD between the two CXL protocols. However, in the anterior corneal layer, this increase was slightly higher with S-CXL. Using in vivo confocal microscopy, Hillenaar et al. showed that keratocyte nuclei are the main source of stromal backscattering of light [25]. Mazzotta et al. reported that the demarcation lines, i.e., the reflection of the transition zone between the anterior, treated stroma and the posterior, untreated stroma, extend deeper with the S-CXL procedure than with the A-CXL protocol ^26^. Accordingly, the CXL-induced influence on densitometry in the anterior region appears to be more pronounced with S-CXL. This could possibly indicate a lower penetration depth with A-CXL.

When performing CXL, resources and thus the applicability of the Bunsen-Roscoe law may be limited. Since intra- and interfibrillar covalent bonds are finite and free reactive amino acid residues of collagen are limited, the cross-linking density can only increase up to its saturation value. Since the superficial corneal layer cannot be cross-linked deeper than the existing attachment points allow, an increase in effect would only be possible in the stromal depth. Oxygen diffusion in the tissue also plays a crucial role. Higher irradiation intensity is associated with higher oxygen consumption, and oxygen deficiency can lead to poor or even absent cross-linking [7, 26, 27]. The result would be a shallower demarcation line depth and thus a smaller cross-linking area [14, 23]. This could lead to a reduced stiffening effect and less topographic flattening after A-CXL than after S-CXL, which some authors have already reported [7, 23, 28]. According to Mazzotta et al., the temporary increase in COD is due to CXL-induced densification and remodeling of stromal collagen [14]. As in previous studies, the present investigation showed that densitometry values return to baseline over time [14, 29]. This may be consistent with the clinical observation of a gradual decrease in corneal haze after CXL. Importantly, there appear to be no long-term differences in the degree of corneal clarity after CXL between the two procedures. Densitometry values normalized in both groups over the long-term course. A smaller initial increase in OD might be achieved by employing the novel technique recently described by Borroni et al., in which riboflavin is delivered into the corneal stroma without epithelial removal by manually creating an epithelial pocket (“Epi-Flap CXL”) [30]. Using this approach, the authors reported significantly less anterior corneal haze (as measured by COD) after 12 months compared with the standard epithelium-off group [31].

In the current study, HOAs coma and RMS coma differed significantly from baseline in both groups at 36 and 96 months. After A-CXL, a significant reduction in RMS anterior and RMS anterior & posterior was also observed at the last two examinations. This result also suggests that HOA changes induced by A-CXL may be more extensive. In line with this, it has already been shown that CXL can lead to a reduction in HOAs [14, 32]. In a sub-analysis, Eslami et al. described an association between HOA reduction and an improvement in corrected distance visual acuity [32]. Higher preoperative aberration was associated with a greater improvement in HOAs, but not with greater topographic flattening. However, since visual acuity improved significantly in these cases, HOAs may be more important for the postoperative development of visual acuity than topographic changes [14, 32]. However, the associations described by Eslami et al. between postinterventional HOA reduction and improvement on visual acuity cannot be confirmed by the present study.

Postinterventional visual acuity remained largely unchanged from baseline after both S-CXL and A-CXL. Only S-CXL showed borderline significant improvement at 8 years. Previous reports on the evolution of visual acuity after CXL are inconsistent. Some authors noted significant improvement [14, 32, 33], while others described postinterventional stability [34, 35]. Similar to the current results, Salman et al. found a trend toward improved visual acuity over a longer period (10 years), but this trend did not reach statistical significance [36]. Prinz et al. also found no significant changes in visual acuity over a two-year observation period [22]. Notably, Mazzotta et al. and Shaheen et al. observed an improvement in visual acuity at the initial measurement points in the first months and one year after CXL, respectively [14, 33]. Here, too, the subsequent course varied: While in the study by Mazzotta and colleagues, postoperative visual acuity remained stable over the years [14], Shaheen et al. found no improvement in visual acuity after 3 and 7 years [35]. In contrast, in the current study, a significant improvement in visual acuity was only observed at the last measurement time point in the S-CXL group. However, this result must be interpreted with caution, because the borderline significance and the different group sizes limit its credibility. In addition, patients in the S-CXL group had poorer preoperative visual acuity, while patients in the A-CXL group were treated at a milder stage of KC. It should be noted, however, that both S-CXL and A-CXL are capable of maintaining at least stable visual acuity over a long postoperative period.

Conventionally, the corneal epithelium is removed at the beginning of CXL treatment, which can lead to postinterventional problems [37]. Pain and persistent epithelial defects, non-infectious and infectious keratitis, corneal opacities and reduced and blurred vision are the most commonly reported complications [37–39]. During the first months after CXL, wound healing processes occur in the cornea, which resolve after approximately 12 months. Therefore, postoperative complications were assessed at follow-up examinations after 12 and 36 months.

Keratitis can also lead to significant scarring and the appearance of infectious and non-infectious corneal infiltrates after CXL is possible. In the present study, only one patient in the A-CXL group developed a keratitis in the early postoperative period. No significant group differences were observed regarding other postinterventional changes. The proportion of patients without clinical postinterventional complications and the incidence of postoperative scars were approximately the same. Corneal opacities appears to be associated with keratocyte loss, corneal edema [37, 38], and corneal remodeling or disease progression [40]. Haze is often transient after CXL, with a reduction 3 to 6 months after the procedure [37, 40]. This mild opacification has been described subepithelially as a result of epithelial debridement [40] and in the anterior stroma as a result of multiple factors [37, 38, 40]. Less commonly, corneal opacities are exaggerated and persistent, leading to corneal scarring due to abnormal wound healing response [40]. Raiskup et al. found stromal opacities in 8.6% of 163 eyes, which persisted during a one-year follow-up [2]. Subsequent permanent (local) scarring is the result of progressive corneal stromal damage and an exaggerated remodeling response [40]. Koller et al. reported stromal scarring in 2.9% of cases after CXL [38]. In the same study, sterile infiltrates were observed in 7.6% of cases. Their resolution may be associated with permanent scarring. Fortunately, infectious post-CXL keratitis is rare and its course varies depending on the etiologic pathogen [37].

As previously mentioned, a flatter demarcation line, thus a smaller cross-linked area and a lesser stiffening effect can be expected with A-CXL [14, 23, 28]. Therefore, one could consider not recommending this procedure in cases of alterations of the most superficial corneal layers, for example, after LASIK (laser-assisted in situ keratomileusis). Shajari et al. suggested preferring A-CXL in patients with thin corneas [23]. However, based on the results of the current study, no differences in the resulting corneal thickness were observed between the two procedures. The clear advantage of A-CXL is the shortened treatment time, which leads to greater patient comfort and more efficient workflow in the clinic.

There are some limitations of the study. As mentioned above, to avoid device switching, a smaller S-CXL group was used, which does not represent the whole patient cohort of our clinic. Therefore, a survival analysis is limited in this study. However, we already published the success rates of S-CXL and A-CXL with a similar follow-up interval demonstrating a non-inferiority for the A-CXL protocol [41]. Furthermore, significant group differences were already evident at baseline. However, since comparisons with baseline values were always performed within the groups, the main results of the study remain. Moreover, deficiencies may arise from the retrospective data analysis. A strength of the study is the very comprehensive data collection over a very long follow-up period.

In summary, the present study demonstrated over a long observation period that both S-CXL and A-CXL can halt the progression of KC. In both groups, significant changes in tomography and densitometry results, as well as HOAs, were observed after CXL compared to baseline. Postinterventional visual acuity development was nearly similar in both groups, but only the S-CXL group showed a borderline significant increase after 8 years. Densitometry values normalized in both groups over the long-term course. Nevertheless, overall, it can be concluded that the A-CXL protocol was non-inferior to the standard procedure. Therefore, S-CXL and A-CXL provide successful and equivalent results in inhibiting KC progression over an 8-year observation period.

## Supplementary Information

Below is the link to the electronic supplementary material.

Supplementary file1 (DOCX 15 KB)

## Data Availability

The dataset used and/or analyzed during the current study is available from the corresponding author on reasonable request.
