# Supplementary material for: Comparison of conventional corneal crosslinking with the accelerated procedure in progressive keratoconus
Source: Int Ophthalmol. 2025 Dec 24;46(1):54. doi: 10.1007/s10792-025-03919-2 (PMC13038651; doi:10.1007/s10792-025-03919-2)
Supplement: Supplementary file 1 — Supplementary file1 (DOCX 15 KB) [file 10792_2025_3919_MOESM1_ESM.docx]

**Supplement 1: Description of the applied CXL protocols.**

| **parameter** |  | **S-CXL group** | **A-CXL group** |
| --- | --- | --- | --- |
| Treatment target |  | Progressive keratoconus | Progressive keratoconus |
| Fluence (total) |  | 5.4 mJ/cm² | 5.4 mJ/cm² |
| Intensity |  | 3 mW/cm² | 9 mW/cm² |
| Treatment time |  | 30 min | 10 min |
| Light source |  | UV-X 1000 | UV-X 2000 |
| Irradiation mode |  | Continuous | Continuous |
| Epithelium status |  | Off (mechanical abrasio) | Off (mechanical abrasio) |
| Chromophore (centration) |  | Riboflavin (0.1 %) | Riboflavin (0.1 %) |
| Chromophore carrier |  | Hydroxypropyl Methylcellulose | Hydroxypropyl Methylcellulose |
